# Relationship between dietary patterns and risk factors for cardiovascular disease in patients with type 2 diabetes mellitus: a cross-sectional study

**DOI:** 10.1186/s12937-016-0132-6

**Published:** 2016-02-04

**Authors:** Yusuke Osonoi, Tomoya Mita, Takeshi Osonoi, Miyoko Saito, Atsuko Tamasawa, Shiho Nakayama, Yuki Someya, Hidenori Ishida, Akio Kanazawa, Masahiko Gosho, Yoshio Fujitani, Hirotaka Watada

**Affiliations:** 1 Department of Metabolism & Endocrinology, Juntendo University Graduate School of Medicine, 2-1-1 Hongo, Bunkyoku, Tokyo 113-8421 Japan; 2 Center for Molecular Diabetology, Juntendo University Graduate School of Medicine, Bunkyoku, Tokyo 113-8421 Japan; 3 Center for Therapeutic Innovations in Diabetes, Juntendo University Graduate School of Medicine, Bunkyoku, Tokyo 113-8421 Japan; 4 Sportology Center, Juntendo University Graduate School of Medicine, Bunkyoku, Tokyo 113-8421 Japan; 5 Naka Memorial Clinic, 745-5, Nakadai, Naka, Ibaraki 311-0113 Japan; 6 Department of Clinical Trial and Clinical Epidemiology, Faculty of Medicine, University of Tsukuba, 1-1-1, Tennodai, Tsukuba, Ibaraki 305-8575 Japan

**Keywords:** Dietary pattern, Type 2 diabetes mellitus, Risk factors for cardiovascular disease, Self-reported questionnaires

## Abstract

**Background:**

While some dietary patterns are associated with the incidence of type 2 diabetes mellitus (T2DM) and cardiovascular disease (CVD), the relationship between dietary pattern and risk factors for CVD in patients with T2DM remains to be clarified. The aim of this study was to identify dietary patterns and investigate the relationship between dietary patterns and potential risk factors for CVD in patients with T2DM.

**Methods:**

The study participants comprised 726 Japanese T2DM outpatients free of history of CVD. Life styles were analyzed using self-reported questionnaires. The relationship between dietary patterns, identified by factor analysis, and potential risk factors for CVD was investigated by linear and logistic regression analyses.

**Results:**

Six dietary patterns were identified by factor analysis. Especially, three dietary patterns were associated with risk factors for CVD. The “Seaweeds, Vegetables, Soy products and Mushrooms” pattern, characterized by high consumption of seaweeds, soy products and mushrooms, was associated with lower use of diabetes medication and healthier lifestyles. The “Noodle and Soup” pattern, characterized by high consumption of noodle and soup was associated with higher body mass index, alanine aminotransferase, aspartate aminotransferase, γ-glutamyl transpeptidase and triglyceride levels. The “Fruit, Dairy products and Sweets” pattern was associated with lower γ-glutamyl transpeptidase levels, blood pressure, albuminuria and brachial-ankle pulse wave velocity.

**Conclusions:**

The findings suggested that dietary patterns correlated with risk factors for CVD in T2DM patients.

## Background

The onset of type 2 diabetes mellitus (T2DM) is associated with numerous lifestyle problems. Indeed, multiple lifestyle modifications, in addition to pharmacological intervention for classical risk factors, can reduce both the incidence of T2DM [1] in non-T2DM population and the development of cardiovascular disease (CVD) in patients with T2DM [2, 3]. However, a recent clinical trial showed that life style intervention, with a special focus on reduced calorie intake and increased physical activity, did not affect the rate of CVD in obese patients with T2DM [4]. In this regard, it is possible that not only caloric intake, but rather dietary composition, may need to be modified to achieve appropriate metabolism and prevent or delay CVD in patients with T2DM.

Recent studies showed that specific foods such as fruit and vegetables [5], nutritional supplements, such as magnesium and calcium [6–8], and quality of carbohydrate intake, such as high fibers, low glycemic index and whole grain intake [8–10], were associated with lower risk of T2DM. However, in real life, people do not consume certain foods or single nutrients, but rather mixed food that contains various nutrients. In this regard, the dietary pattern should be taken into consideration, because it reflects the complexity of dietary intake, where the food contents usually have interactive and synergistic effects and occasionally even antagonistic effects [11]. In fact, it was suggested that dietary patterns may be more predictive of disease risks compared to specific food- and nutrient-based approaches [11].

Recent studies demonstrated that healthy Asian dietary patterns characterized by high consumption of vegetables, fruits, seaweeds, bonefishes, potatoes and/or soy foods were inversely associated with incidence of T2DM [12–14], metabolic syndrome [15], atherosclerosis [16] and related CVD [17] in non-T2DM general population, while western dietary pattern was associated with high incidence of T2DM [10, 18, 19] and metabolic syndrome [20]. Although it was demonstrated that the consumption of fruits, vegetable foods and meat was higher in patients with T2DM than non-T2DM subjects [21], only a few studies demonstrated that Korean Healthy diet pattern characterized by whole grains, legumes, vegetables and fruits was inversely associated with lipid metabolism [22], and vegetable and fish pattern was associated with better renal function in patients with T2DM [23]. Thus, there seems to be some gaps in our understanding of the relationship between dietary patterns and risk factors for CVD, especially arterial stiffness, in patients with T2DM. The aim of this cross-sectional study was to assess the above relationship in Japanese patients with T2DM free of history of CVD

## Research design and methods

### Subjects

The subjects of this cohort study were recruited from the Diabetes Outpatient Clinic of Juntendo University (Tokyo, Japan), Naka Memorial Clinic (Naka, Japan), and Secomedic Hospital (Funabashi, Japan) as reported previously [24]. Briefly, the inclusion criteria were as follows: *1)* T2DM patients, *2)* ≥25 years of age and <70 years of age (regardless of gender), and *3)* signing consent form for participation in the study. The following exclusion criteria were also applied: *1)* type 1 or secondary diabetes, *2)* presence of severe infectious disease, before or after surgery, or severe trauma, *3)* history of myocardial infarction, angina pectoris, cerebral stroke, or cerebral infarction, *4)* chronic renal failure requiring hemodialysis, *5)* liver cirrhosis, *6)* moderate or severe heart failure (NYHA/New York Heart Association stage III or higher), *7)* active malignancy, *8)* pregnant, lactating, or possibly pregnant women, or those planning to become pregnant during the study period, *9)* patients judged as ineligible by the clinical investigators.

A total of 1,032 consecutive subjects were screened between June 2013 and January 2014. Among them, 906 patients who met the above eligibility criteria were invited to participate in the present study. After providing information on the purpose and procedures of the study, 736 patients with T2DM accepted the invitation and were enrolled in this study. The study was approved by the Institutional Review Board of Juntendo University Hospital and conducted in accordance with the principles described in the Declaration of Helsinki. All patients provided written informed consent prior to participation. The study was registered on the University Hospital Medical Information Network Clinical Trials Registry (UMIN000010932).

### Questionnaire survey

Questionnaire survey was conducted using valid and reliable self-administered questionnaires described previously [24]. Briefly, dietary habits during the preceding month were assessed with the validated, Brief, self-administered Diet History Questionnaire (BDHQ). The BDHQ is a 4-page structured questionnaire that asks about consumption and frequency of selected foods to estimate the dietary intake of 56 food and beverage items with specified serving size described in terms of consumption in general Japanese populations [25].

We also used the Morning Evening Questionnaire (MEQ) [26], which is a self-assessment questionnaire developed primarily for screening candidates for sleep-related experiments to evaluate morningness and eveningness in individuals. A high MEQ score represents morning type.

The Pittsburg Sleep Quality Index (PSQI) [27] is a self-administered questionnaire designed to evaluate sleep quality and consists of 18 items that in turn are comprised of 7 components, which include subjective sleep quality, sleep duration, sleep onset, habitual sleep efficiency, sleep disturbances, use of sleeping medications, and daytime dysfunction, with each weighted equally on a 0–3 scale, to be summed to yield the global PSQI score ranging from 0 to 21, where the higher the scores, the worse the sleep quality. The PSQI has a high test-retest reliability and a good validity [28].

The participating patients also completed the BDI (Beck Depression inventory)-II, which is a 21-item questionnaire that assesses hopelessness, irritability, cognition, guilt, fatigue, weight loss, and sexual interest, representing depression-related symptoms in adults and adolescents [29]. A high BDI-II score represents depressive state.

Physical activity level was assessed with the International Physical Activity Questionnaire (IPAQ) that comprises four simple questions on physical activity [30]. The IPAQ results are expressed as metabolic equivalent scores (METs-hour-week^−1^).

Workers were defined as full-time employees or shift workers by a question in the questionnaire, as described previously [24]. The subjects were also divided into non-smokers, former smokers or current smokers, as described previously [24].

### Blood and urine tests

Blood samples were obtained at visits to the Outpatients Clinic after overnight fast. Liver and renal function tests, lipids, HbA1c (National Glycohemoglobin Standardization Program), and glucose were measured with standard techniques. UAE was measured by the latex agglutination assay using a spot urine sample. The estimated glomerular filtration rate (eGFR) was calculated by the formula: eGFR (ml/min per 1.73 m^2^) =194× Age^-0.287^× serum creatinine^-0.1094^ (×0.739 for females), as described previously [24] .

### Measurement of baPWV

baPWV was measured using an automatic waveform analyzer (BP-203RPE; Colin Medical Technology, Komaki, Japan), as described previously [24, 31]. Briefly, recording was performed with the patients in the supine position after resting for five minutes. Occlusion and monitoring cuffs were placed snugly around both areas in the upper and lower extremities. The pressure waveforms were then recorded simultaneously from the brachial arteries by the oscillometric method. All scans were automatically conducted by well-trained investigators who were blinded to the clinical information. The validity and reproducibility of baPWV measurements have been confirmed to be considerably high [32].

### Statistical analysis

Results are presented as mean ± SD or median (interquartile range: 25 % to 75 %) for continuous variables or number (proportion) of patients for categorical variables. Some parameters were logarithmically transformed to approximate normal distribution. We used factor analysis with varimax rotation to reduce the complexity of dietary patterns of patients with T2DM based on the results of BDHQ. Factors with an eigenvalue >1.25 were retained [33]. Individual food items with a factor loading of > |0.4| are highlighted as composing that factor for simplicity. The factor scores for each dietary pattern and for each subject were calculated by summing each dietary pattern score weighted by their factor loadings. The estimated factor scores were categorized into quintiles. Trend association across the quintile was evaluated by linear regression analysis for continuous variables or logistic regression analysis for categorical variables. We developed three models to evaluate the trend adjusted for age and gender or adjusted for age, gender, and body mass index (BMI), MEQ, PSQI, BDI-II, current smoking and physical activity. The model for eGFR was adjusted for BMI, MEQ, PSQI, BDI-II, current smoking and physical activity. Statistical tests were two-sided with 5 % significant level. All analyses were performed using the SAS software version 9.3 (SAS Institute, Cary, NC).

## Results

Among 736 patients, 10 patients did not complete the questionnaires, and were thus excluded from analysis. The study participants comprised 726 Japanese patients with T2DM who were being treated on an outpatient basis. Table 1 lists the characteristics of the study subjects. The mean age was 57.8 ± 8.6 years, 62.9 % male and HbA1c was 7.0 ± 1.0 %.

**Table 1:** Patients characteristics (*n* = 726)

Demographic data	
Age (years)	57.8 ± 8.6
Gender (male)	456 (62.9)
Estimated duration of diabetes (years)	9.9 ± 7.2
Body mass index (kg/m^2^)	24.6 ± 4.1
HbA1c (%)	7.0 ± 1.0
Fasting blood glucose (mg/dl)	134 ± 31
Systolic blood pressure (mmHg)	127 ± 14
Diastolic blood pressure (mmHg)	77 ± 11
Total cholesterol (mg/dL)	185 ± 28
High-density lipoprotein-cholesterol (mg/dL)	59 ± 14
Triglyceride (mg/dL)	100 [70, 152]
Aspartate aminotransferase (U/L)	21 [18, 27]
Alanine aminotransferase (U/L)	22 [16, 33]
γ-glutamyl transpeptidase (U/L)	25 [17, 39]
Uric Acid (mg/dl)	5.5 ± 1.2
Estimated glomerular filtration rate (ml/min/ 1.73 m^2^)	78 ± 18
Urinary albumin excretion (mg/g creatinine)	10 [6, 23]
brachial-ankle pulse wave velocity (cm/s)	1543 ± 279
Morningness-Eveningness Questionnaire	57.4 ± 7.3
Pittsburg Sleep Quality Index	5.1 ± 3.0
Beck Depression inventory -II	9.9 ± 7.6
Energy intake (kcal/day)	1713 ± 582
Physical activity (Mets/h/week)	42.8 ± 70.5
Sleep duration (hours)	6.4 ± 1.2
Current smoker (yes)	174 (24.0)
Alcohol (g/day)	12.3 ± 21.5
Treatment modality (n/%)	
Diabetes medication (yes)	620 (85.5)
Hypertension medication (yes)	346 (47.7)
Hyperlipidemia medication (yes)	442 (61.0)

Data are mean ± SD or number (percentage) of patients

Factor analysis with varimax rotation identified six life style patterns (Table 2). Factor 1, with high loading for seaweeds, vegetables, soy products and mushroom, was labeled the “Seaweeds, Vegetables, Soy products and Mushrooms” pattern. Factor 2, which was characterized by fish, potatoes, meat and fats and oils, was labeled “Fish and Meat” pattern. Factor 3 seemed to load consumption of noodle and soup and thus it was named “Noodle and Soup” pattern. Factor 4 seemed to be characterized by meat, fats and oils, seasonings and eggs, and was named “Meat, Fats and Oils, Seasonings and Eggs” pattern. Factor 5 seemed to be characterized by sweet, fruit and dairy products and was labeled “Fruit, Dairy products and Sweets” pattern. Factor 6 seemed to be characterized by rice and miso soups, and was named “Rice and Miso soups” pattern. Overall, these six diet patterns could account for 48 % of the variance in food intake.

**Table 2:** Food items and factor analysis with varimax rotation

Components and item labels	Factor 1	Factor 2	Factor 3	Factor 4	Factor 5	Factor 6
Rice	−0.07	0.07	0.00	0.06	−0.15	**0.80**
Noodle	0.07	0.14	**0.96**	0.04	0.02	0.05
Breads	0.07	−0.07	0.24	0.36	**0.42**	−0.15
Miso soup	0.18	0.02	0.08	−0.06	0.09	**0.81**
Dairy products	0.27	−0.07	−0.01	0.17	**0.45**	0.06
Meats	0.03	**0.52**	0.06	**0.52**	−0.09	0.02
Processed meats	0.12	0.02	0.01	**0.66**	0.16	−0.03
Fish and shellfish	0.32	**0.63**	0.17	0.16	−0.03	0.05
Processed fish	0.23	**0.60**	0.10	0.16	0.07	0.14
Eggs	0.20	0.32	0.01	**0.41**	0.03	−0.01
Soy products	**0.63**	0.10	0.13	0.13	0.13	0.08
Green & dark yellow vegetables	**0.74**	0.07	−0.11	0.29	0.06	−0.07
White vegetables	**0.73**	0.25	0.11	0.13	0.00	−0.07
Pickled vegetables	**0.51**	0.18	−0.03	−0.07	0.04	0.10
Fruit and vegetable Juices	0.04	0.16	−0.01	−0.01	0.02	−0.09
Fruit	0.21	0.35	−0.01	−0.03	**0.56**	−0.05
Sugary foods	0.15	**0.51**	0.00	−0.05	0.20	0.21
Mushrooms	**0.55**	**0.41**	0.01	0.01	0.13	−0.14
Seaweeds	**0.70**	0.06	0.07	0.05	−0.02	0.10
Potatoes	0.18	**0.62**	0.01	0.03	0.21	−0.07
Sweets	−0.03	0.21	0.13	0.17	**0.62**	0.06
Fats and oils	−0.07	**0.49**	0.22	**0.64**	−0.08	0.02
Alcohol	0.01	0.02	0.20	0.17	**−0.53**	0.15
Tea	0.04	0.06	0.03	0.13	0.34	0.11
Coffee	0.09	0.02	0.04	0.14	0.14	0.01
Soft drinks	−0.05	−0.09	0.21	0.19	0.07	0.03
Seasonings	0.32	0.05	−0.09	**0.60**	0.03	0.06
Soup	0.08	0.11	**0.96**	0.02	0.01	0.04
Contribution	19 %	8 %	6 %	6 %	5 %	4 %

Individual food items with a factor loading of >|0.4| are shown in bold

The characteristics across quintile of dietary pattern are shown in Tables 3, 4, 5 and 6. Table 3 shows the characteristics of the study subjects according to the quintiles of “Seaweeds, Vegetables, Soy products and Mushrooms” pattern scores in age and gender adjusted model. Subjects with a higher score for this pattern were older, more morning type, higher consumption of food, higher physical activity and less depressive status. While HbA1c levels were comparable between the lowest quintile and the highest quintile, the lower prevalence of diabetes medication was found in the highest quintile in age- and gender-adjusted model. Subjects with a higher score for this pattern had lower aspartate aminotransferase (AST), γ-glutamyl transpeptidase (γ-GTP) and UAE values although these findings disappeared in multivariable adjusted model. Subjects with a higher score for “Fish and Meat” pattern were likely to be more female and higher consumption of food intake (Table 3). On the other hand, there were no significant trend in risk factors for CVD among quintiles in both age and gender, and multivariable adjusted model (Table 5). Table 3 also shows the characteristics of the study subjects according to the quintiles of “Noodle and Soup” pattern scores. Subjects with a higher score for this pattern were likely to be males with higher BMI and consumption of food intake. Furthermore, patients with a higher score of this pattern had significantly higher AST, alanine aminotransferase (ALT) levels and γ-GTP compared to those with a lower score in both age- and gender-adjusted model, and multivariate adjusted model (Table 5). Furthermore, patients with a higher score of this pattern had significantly higher triglyceride levels compared to those with a lower score in the age- and gender-adjusted model.

**Table 3:** Characteristics according to quintile categories based on dietary patterns

Variable	Seaweeds, vegetables, soy products and mushrooms			Fish and meat			Noodle and soups		
	Quintile 1	Quintile 5	Model 1	Quintile 1	Quintile 5	Model 1	Quintile 1	Quintile 5	Model 1
Age (years)	53.9 ± 8.9	59.5 ± 8.2	-	58.3 ± 8.6	57.9 ± 8.9	-	59.1 ± 7.9	57.6 ± 8.7	-
Gender (male)	92 (63.4)	83 (57.2)	-	108 (74.5)	86 (59.3)	-	83 (44.4)	133 (85.3)	-
Body mass index (kg/m^2^)	25.8 ± 4.0	24.1 ± 4.2	−1.64	24.7 ± 3.8	24.8 ± 4.3	0.40	23.9 ± 3.9	25.1 ± 4.1	3.04**
Estimated duration of diabetes (years)	9.5 ± 6.8	9.4 ± 6.4	−1.44	9.9 ± 6.8	10.4 ± 6.9	1.00	9.7 ± 7.3	9.5 ± 7.0	0.00
MEQ	55.3 ± 8.0	58.7 ± 6.8	2.55***	57.6 ± 7.5	57.6 ± 7.4	−0.11	57.9 ± 7.4	57.5 ± 7.1	−1.21
PSQI	5.8 ± 3.8	4.8 ± 2.6	−1.53	5.0 ± 3.1	5.5 ± 3.4	1.35	5.1 ± 3.4	5.1 ± 2.8	1.03
BDI-II	11.4 ± 7.5	9.1 ± 7.4	−2.18*	10.0 ± 7.5	10.8 ± 8.0	0.71	9.8 ± 8.1	9.6 ± 7.9	0.47
Energy intake (kcal/day)	1614 ± 655	2000 ± 546	6.43***	1621 ± 506	2154 ± 658	10.90***	1542 ± 502	2055 ± 665	7.55
Current smoker (%)	34 (23.4)	27 (18.6)	−0.28	41 (28.3)	27 (18.6)	−0.64	42 (22.5)	46(29.5)	0.14
Physical activity (kcal/day)	37.2 ± 65.0	51.7 ± 84.1	2.17*	44.5 ± 65.1	46.2 ± 85.6	1.14	40.3 ± 67.9	51.8 ± 95.2	1.73
Worker (yes)	114 (78.6)	92 (63.4)	−1.22	105 (72.4)	105 (72.4)	1.31	132(70.6)	118 (75.6)	−0.84
Sleep duration (hours)	6.4 ± 1.4	6.5 ± 1.2	−1.15	6.5 ± 1.3	6.4 ± 1.3	−0.14	6.4 ± 1.3	6.4 ± 1.1	−0.27
Diabetes medication (yes)	130 (89.7)	114 (78.6)	−2.50*	122 (84.1)	120 (82.8)	−033	155 (82.9)	132 (84.6)	−0.21
Hypertension medication (yes)	74 (51.0)	62 (42.8)	−1.84	71 (49.0)	78 (53.8)	1.60	85 (45.5)	74 (47.4)	−0.03
Hyperlipidemia medication (yes)	90 (62.1)	87 (60.0)	−0.93	89 (61.4)	86 (59.3)	−1.10	106 (56.7)	86 (55.1)	1.37

Data are mean ± SD, median [range: 25 % to 75 %] or number of subjects (percentage) before adjustment. *P < 0.05, **P < 0.01, ***P < 0.001

Model 1: Trend estimation for linear trends across quintiles is based on linear regression analysis for continuous variables or logistic regression analysis for categorical variables adjusted for age and gender. Standardized regression coefficients are shown. *BDI* beck depression inventory, *MEQ* morningness-eveningness questionnaire, *PSQI*: Pittsburg sleep quality index

**Table 4:** Characteristics according to quintile categories in each dietary pattern

Variable	Meat, fats and oils, seasonings and eggs			Fruit, dairy products and sweets			Rice and miso soups		
	Quintile 1	Quintile 5	Model 1	Quintile 1	Quintile 5	Model 1	Quintile 1	Quintile 5	Model 1
Age (years)	60.6 ± 6.7	54.9 ± 8.9	-	56.6 ± 9.1	58.7 ± 8.2	-	57.5 ± 8.8	58.3 ± 8.6	-
Gender (male)	104 (55.3)	132 (72.9)	-	134 (82.7)	109(55.9)	-	106 (55.5)	137 (76.5)	-
Body mass index (kg/m^2^)	24.3 ± 3.9	25.3 ± 4.5	−0.11	24.4 ± 3.8	24.6 ± 4.2	1.20	25.2 ± 4.3	24.4 ± 3.8	−1.80
Estimated duration of diabetes (years)	10.0 ± 7.4	9.5 ± 6.5	0.47	9.4 ± 7.0	10.2 ± 7.8	0.76	9.5 ± 6.7	10.2 ± 7.0	0.83
MEQ	58.9 ± 7.4	56.5 ± 7.4	−2.14*	58.6 ± 7.8	56.3 ± 7.2	−3.36***	57.1 ± 7.9	56.9 ± 7.5	−0.91
PSQI	4.5 ± 2.6	5.3 ± 3.1	2.00*	5.1 ± 3.0	5.2 ± 3.1	0.02	5.2 ± 2.9	5.0 ± 2.4	−0.06
BDI-II	9.6 ± 8.1	10.0 ± 6.8	0.65	9.7 ± 7.1	10.6 ± 8.6	1.24	10.3 ± 7.9	10.4 ± 8.5	0.98
Energy intake (kcal/day)	1440 ± 479	2118 ± 651	12.94***	1781 ± 618	1909 ± 626	4.14***	1418 ± 456	2047 ± 568	11.01***
Current smoker (%)	39 (20.7)	50 (27.6)	−0.03	55 (34.0)	32 (16.4)	−2.54*	46 (24.1)	42 (23.5)	−1.00
Physical activity (kcal/day)	39.3 ± 65.3	39.1 ± 73.5	0.13	46.4 ± 78.9	45.5 ± 80.7	−0.45	41.3 ± 62.8	47.8 ± 84.4	1.08
Worker (yes)	128 (68.1)	150(82.9)	0.11	134 (82.7)	128 (65.6)	−2.51*	131 (68.6)	138 (77.1)	1.91
Sleep duration (hours)	6.7 ± 1.2	6.3 ± 1.2	−1.88	6.5 ± 1.3	6.5 ± 1.1	−0.02	6.5 ± 1.1	6.4 ± 1.2	−0.55
Diabetes medication (yes)	164(87.2)	151 (83.4)	−0.58	140 (86.4)	164 (84.1)	−1.12	167 (87.4)	153 (85.5)	−0.80
Hypertension medication (yes)	92 (48.9)	82 (45.3)	−0.75	95 (58.6)	82 (42.1)	−2.86**	94 (49.2)	95 (53.1)	0.01
Hyperlipidemia medication (yes)	122(64.9)	99 (54.7)	−0.35	78 (48.1)	131 (67.2)	2.86**	118 (61.8)	95 (53.1)	−1.14

Data are mean ± SD, median [range: 25 % to 75 %] or number of subjects (percentages) before adjustment. *P < 0.05, **P < 0.01, ***P < 0.001

Model 1: Trend estimation for linear trends across quintiles is based on linear regression analysis for continuous variables or logistic regression analysis for categorical variables adjusted for age and gender. Standardized regression coefficients are shown. See Table 3 for abbreviations

**Table 5:** Cardio-renal-metabolic parameters according to quintile categories in each dietary pattern

Variable	Seaweeds, vegetables, soy products and mushrooms				Fish and meat				Noodle and soup			
	Quintile 1	Quintile 5	Model 1	Model 2	Quintile 1	Quintile 5	Model 1	Mode l 2	Quintile 1	Quintile 5	Model 1	Model 2
AST (U/L)	22 [19, 29]	22 [17, 26]	−2.07*	−1.71	21 [18, 27]	23 [19, 28]	1.14	0.98	21 [17, 25]	23 [18, 29]	3.25**	2.60*
ALT (U/L)	24 [17, 41]	22 [16, 33]	−1.52	−0.80	23 [16, 34]	25 [17, 34]	0.94	0.69	19 [15, 30]	26 [18, 39]	4.17***	3.29*
γ-GTP (U/L)	28 [18,48]	22 [15, 36]	−2.46*	−1.95	25 [18, 44]	25 [18, 38]	0.13	−0.01	22 [16, 30]	30 [21, 50]	3.62***	2.75**
Uric Acid (mg/dl)	5.5 ± 1.2	5.4 ± 1.2	0.12	0.51	5.6 ± 1.2	5.4 ± 1.2	0.75	0.63	5.2 ± 1.2	5.8 ± 1.2	0.85	0.27
eGFR (ml/min/ 1.73 m^2^)	81 ± 18	77 ± 16	-	0.17	77 ± 18	78 ± 17	-	0.08	78 ± 19	79 ± 17	-	−0.39
Total cholesterol (mg/dl)	190 ± 27	186 ± 29	−1.64	−1.70	184 ± 30	187 ± 27	0.16	0.09	188 ± 30	185 ± 27	0.39	0.22
HDL-C (mg/dl)	59 ± 14	62 ± 14	1.08	0.60	58 ± 13	59 ± 14	−0.03	−0.07	62 ± 15	57 ± 13	−1.39	−0.59
Triglycerides (mg/dl)	101 [71,163]	96 [64, 144]	−1.74	−1.06	94 [66, 144]	112 [67, 152]	0.88	0.78	89 [64, 135]	116 [78, 164]	2.82**	1.73
Fasting blood glucose (mg/dl)	137 ± 34	131 ± 27	0.01	0.49	133 ± 32	138 ± 35	1.01	0.81	132 ± 31	137 ± 30	0.72	0.43
HbA1c	7.1 ± 1.1	6.9 ± 1.1	−0.48	−0.04	6.9 ± 0.9	7.1 ± 1.1	1.02	0.95	6.9 ± 1.0	7.0 ± 1.0	1.66	1.21
Systolic BP(mmHg)	128 ± 13	127 ± 15	0.12	0.83	127 ± 14	126 ± 15	0.09	−0.10	126 ± 15	128 ± 13	1.83	0.92
Diastolic BP (mmHg)	79 ± 10	75 ± 10	−1.00	−0.53	78 ± 13	76 ± 11	−0.19	−0.33	75 ± 14	78 ± 10	0.99	0.27
UAE (mg/g creatinine)	10 [6, 21]	10 [5, 17]	−2.12*	−1.74	10 [6, 24]	10 [7, 23]	−0.80	−0.88	11 [6, 30]	9 [6, 19]	−0.39	−1.18
baPWV (cm/s)	1528 ± 294	1556 ± 262	−1.62	−1.53	1536 ± 258	1535 ± 248	0.79	0.72	1549 ± 288	1536 ± 262	−0.91	−0.94

Data are mean ± SD, median [range: 25 % to 75 %] or number of subjects (percentage) before adjustment. *P < 0.05, **P < 0.01, ***P < 0.001

Model 1: Trend estimation for linear trends across quintiles is based on linear regression analysis for continuous variables or logistic regression analysis for categorical variables adjusted for age and gender. Model 2: Trend estimation for linear trends across quintiles is based on linear regression analysis for continuous variables or logistic regression analysis for categorical variables adjusted for age, gender, BMI, morningness-eveningness questionnaire, Pittsburg Sleep Quality Index, Beck Depression inventory, current smoking, and physical activity. Standardized regression coefficients are shown. *ALT* alanine aminotransferase, *AST* aspartate aminotransferase, *baPWV* brachial-ankle pulse wave velocity, *BP* blood pressure, *eGFR* estimated glomerular filtration rate, *HDL-C* high-density lipoprotein-cholesterol, *UAE* urinary albumin excretion, *γ-GTP* γ-glutamyl transpeptidase

**Table 6:** Cardio-renal-metabolic parameters according to quintile categories in each dietary pattern

Variable	Meat, fats and oils, seasonings and eggs				Fruit, dairy products and sweets				Rice and miso soups			
	Quintile 1	Quintile 5	Model 1	Model 2	Quintile 1	Quintile 5	Model 1	Model 2	Quintile 1	Quintile 5	Model 1	Model 2
AST (U/L)	22 [19, 27]	21 [17, 26]	−1.14	−1.15	22 [18, 28]	21 [18, 27]	0.49	0.09	21 [17, 27]	22[18, 27]	1.05	1.45
ALT (U/L)	21 [16, 31]	24 [17, 35]	−0.40	−0.49	22 [16, 33]	23 [17, 34]	2.58*	2.10*	22 [15, 35]	24 [17, 34]	0.92	1.48
γ-GTP (U/L)	25 [16, 36]	27 [17, 46]	−1.19	−1.33	32 [20, 56]	24 [17, 36]	−3.51***	−4.20***	26 [17, 38]	26 [19, 42]	0.11	0.69
Uric Acid (mg/dl)	5.4 ± 1.2	5.5 ± 1.2	−2.27*	−2.31*	5.8 ± 1.3	5.3 ± 1.2	−0.58	−1.16	5.4 ± 1.3	5.6 ± 1.1	0.23	0.46
eGFR (ml/min/ 1.73 m^2^)	75 ± 15	80 ± 19	-	1.24	78 ± 17	79 ± 19	-	−0.56	79 ± 19	77 ± 17	-	−0.87
Total cholesterol (mg/dl)	185 ± 26	188 ± 30	0.79	0.83	185 ± 27	185 ± 29	−1.04	−1.11	188 ± 29	183 ± 29	−0.26	−0.09
HDL-C (mg/dl)	59 ± 14	59 ± 16	1.25	1.35	61 ± 14	60 ± 14	−2.87**	−2.65**	59 ± 15	58 ± 13	−0.50	−1.05
Triglycerides (mg/dl)	98 [68,144]	112 [72, 157]	−0.14	−0.27	112 [70, 158]	98 [71, 143]	−0.45	−0.99	99 [71, 148]	100 [70, 158]	−0.07	0.70
Fasting blood glucose (mg/dl)	130 ± 30	137 ± 33	0.93	0.64	136 ± 30	130 ± 31	−1.22	−1.74	135 ± 33	136 ± 31	0.28	0.21
HbA1c	6.8 ± 0.9	7.0 ± 1.0	1.55	1.25	6.8 ± 1.0	6.9 ± 0.9	0.56	0.12	7.0 ± 1.1	7.0 ± 0.9	0.65	0.79
Systolic BP(mmHg)	126 ± 15	127 ± 13	0.37	0.22	128 ± 13	125 ± 14	−2.65**	−3.01**	128 ± 15	127 ± 13	−0.47	0.10
Diastolic BP (mmHg)	76 ± 10	79 ± 13	−0.37	−0.44	80 ± 11	76 ± 12	−1.89	−2.31*	77 ± 11	78 ± 13	1.19	1.61
UAE (mg/g creatinine)	10 [6, 24]	10 [5, 24]	−0.17	−0.28	12[7, 36]	9 [6, 18]	−3.20**	−3.44***	10 [6, 23]	11 [6, 23]	−1.23	−0.77
baPWV (cm/s)	1577 ± 282	1508 ± 265	−0.07	−0.33	1574 ± 314	1530 ± 259	−3.04**	−3.14**	1536 ± 256	1556 ± 277	0.18	0.15

Data are mean ± SD, median [range: 25 % to 75 %] or number of subjects (proportion) before adjustment. *P < 0.05, **P < 0.01, ***P < 0.001

Model 1: Trend estimation for linear trends across quintiles is based on linear regression analysis for continuous variables or logistic regression analysis for categorical variables adjusted for age and gender. Model 2: Trend estimation for linear trends across quintiles is based on linear regression analysis for continuous variables or logistic regression analysis for categorical variables adjusted for age, gender, BMI, morningness-eveningness questionnaire, Pittsburg Sleep Quality Index, Beck Depression inventory, current smoking, and physical activity. Standardized regression coefficients are shown. See Table 5 for abbreviations

Table 4 shows the characteristics of the study subjects according to the quintiles of “Meat, Fats and oils, Seasonings and Eggs” pattern scores in age- and gender-adjusted model. Subjects with a higher score for this pattern were younger males with lower score of MEQ, higher score of PSQI and higher consumption of food intake. On the other hand, there were no significant trend in risk factors for CVD except uric acid in both age- and gender-adjusted model and multivariable adjusted model (Table 6). Table 4 also displays the characteristics of the study subjects according to the quintiles of “Fruit, Dairy products and Sweets” pattern scores. This pattern was also characterized by lower consumption of alcohol. Subjects with a higher score for this pattern were more likely non-worker, non-smoker females with higher consumption of food intake, with lower score of MEQ. Lower use of hypertension medication and higher use of hyperlipidemia mediation were found in the highest quintile. Regarding risk factors for CVD, patients with higher a score of this pattern had lower γ-GTP level, HDL level, systolic BP, UAE and baPWV but slightly higher ALT level compared to those with a lower score in age- and gender-adjusted model, and multivariate adjusted model (Table 6). Table 4 shows the characteristics of the study subjects according to the quintiles of “Rice and Miso soups” pattern scores. Subjects with a higher score for this pattern were higher consumption of food while there was no significant trend in risk factors for CVD.

## Discussion

Among the six dietary patterns classified in this study, the “Seaweeds, Vegetables, Soy products and Mushrooms” pattern was associated with lower use of diabetes medications and healthier lifestyles. “Noodle and soup” pattern was associated with higher BMI, higher AST, ALT, γ-GTP and triglyceride levels. “Fruit, Dairy products and Sweets” pattern was associated with lower γ-GTP levels, BP, UAE and baPWV. On the other hand, the remaining three dietary patterns did not negatively affect risk factors for CVD.

Subjects with a higher score of “Seaweeds, Vegetables, Soy products and Mushrooms” pattern had healthier lifestyles, such as more morning type, less depressive symptoms and higher physical activity. Even after adjustment for those factors, the “Seaweeds, Vegetables, Soy products and Mushroom” pattern was associated with lower use of diabetes medication (data not shown), while there was no significant trend of HbA1c among their quintile categories. It is possible that the “Seaweeds, Vegetables, Soy products and Mushrooms” pattern positively affect glucose metabolism even in patients with T2DM, since a dietary pattern characterized by high consumption of vegetables, soy products and seaweed was reported to be inversely associated with lower incidence of T2DM [12–14] in the general population. Fiber [8], antioxidant vitamins [34], magnesium [6, 8] and phytoestrogens [35], which were abundant in those foods, have been reported to improve glucose metabolism and reduce insulin resistance. In addition, one previous study demonstrated that a healthy Japanese dietary pattern characterized by high consumption of vegetables, fruit, mushrooms and soy products was associated with fewer depressive symptoms in municipal employees [36]. Consistent with this finding, the almost similar type of “Seaweeds, Vegetables, Soy products and Mushrooms” pattern defined in this study was also associated with lower prevalence of depressive symptoms in patients with T2DM. Generally, vegetables and mushrooms contain vitamins and folate, which may reduce the prevalence of depression. In fact, it was demonstrated that antioxidant vitamins may protect neuropsychiatric disorders [37]. Similarly, folate intake was reported to be reduce depressive symptoms through metabolism of monoamines like serotonin and homocysteine in the brain [38]. Taken together, it seems that “Seaweeds, Vegetables, Soy products and Mushroom” dietary pattern is beneficial for patients with T2DM.

The “Fish and Meat” pattern was not associated with risk factors for CVD in this study. It was demonstrated that similar dietary pattern was also associated with the prevalence of the metabolic syndrome in Western populations [39] but not in Japanese workers [40]. These distinct results may be associated with relatively lower intake of meat and higher consumption of fish, vegetable and fruit in Japanese people compared to those in Western populations.

A higher score of “Noodle and Soup” pattern was associated with obesity characterized by high BMI, high AST, ALT, γ-GTP and triglyceride levels. Noodles including Japanese noodles, Chinese style noodles and pasta are one of the most favorite dishes in Japan. Higher intake of noodles, which contains high carbohydrate, may lead to obesity and fatty liver. Higher intake of oil included in the soup may be also associated with obesity and fatty liver.

We found that “Meat, Fats and Oils, Seasonings and Eggs” pattern represented by high intake of meat, fats and oils, seasonings and eggs was not related with risk factors for CVD, except high uric acid levels. On the other hand, a recent study reported that a similar dietary pattern, named high-fat pattern, was inversely associated with HbA1c levels in Japanese non-T2DM population [41]. The difference between our study and the above could be related to a number of differences among subjects, such as T2DM or non-T2DM, obesity, fat-derived energy intake and other dietary components. While frequent intake of meat, processed meat and eggs negatively affect lipid metabolism, “Meat, Fats and Oils, Seasonings and Eggs” pattern was not associated with lipid metabolism in this study. This may be related to the relatively higher intake of vegetables in addition to frequent intake of meat, processed meat and eggs. Indeed, it was reported that vegetable-rich diet seems to reduce cholesterol concentrations and other cardiovascular risk factors in patients with T2DM as effectively as in patients with non-T2DM [42].

The “Fruit, Dairy products and Sweets” pattern with less frequent consumption of alcohol and cigarette seemed to be preferred by females. Especially, this pattern was associated with lower BP, UAE and baPWV, even after adjustment for multivariate factors. These data may be reasonable because frequent dietary fruit intake was shown to be associated with lower BP and reduced atherosclerotic changes [43]. Also, previous studies demonstrated favorable preventive effects of dairy product on the incidence of hypertension, atherosclerosis and T2DM [44]. Antioxidant nutrients, such as vitamin C and E, carotene and dietary fiber are abundant in fruit. Similarly, dairy products contain calcium, magnesium, potassium, and vitamin D. These nutrients may have beneficial roles on risk factors for CVD in this pattern despite the co-presence of unhealthy diet pattern such as higher sweet consumption.

Recent studies showed that high consumption of rice contributed to the onset of T2DM in Chinese [9] and Japanese women, but not Japanese men [45]. While the mechanism by which frequent intake of rice increases the risk of T2DM remains largely unknown, the high glycemic index of white rice may affect glucose metabolism [46]. However, the “Rice and Soups” pattern was not associated with glycemic control in our patients with T2DM. Considering that subjects with high scores of this pattern showed higher consumption of fiber (data not shown), they may consume brown rice, which contains more dietary fiber, vitamin and minerals compared with white rice or fiber-rich foods with rice.

The present study has certain limitations. First, the cross-sectional design does not allow inference of a causal relationships between dietary patterns and risk factors for CVD. In addition, we could not confirm that the participating subjects adhered to the same dietary pattern throughout life. Especially, dietary patterns probably change depending on many factors such as status of glycemic control (good glycemic control vs. poor glycemic control) and estimated duration of T2DM (shorter duration vs. longer duration). This may cause reverse causality. Thus, it is not reasonable to conclude that our data could be generalized to a wider range of population, even in Japanese patients with T2DM. Second, we evaluated dietary pattern by self-reported questionnaires, though this method has been widely used in many studies. The results may be influenced by social desirability and recall bias. In addition, we evaluated only 56 food and beverage items. This could be associated with incomplete list of dietary patterns and an underestimation of energy intake. Finally, our results may be affected by several limiting features of factor analysis. It is not possible for investigators to avoid making judgmental decision at their discretion [47]. The results are dependent on the criterion to determine the number of factors to be retained and, the methods for rotation of factor axes and for labeling the dietary pattern. Therefore, our data should be interpreted with caution. For example, other remaining dietary patters or other remaining food items in each dietary pattern may be associated with risk factors for CVD.

## Conclusion

Our study showed the relationship between dietary patterns and risk factors for CVD in T2DM patients without history of CVD. Thus, dietary pattern could be a potentially important therapeutic target to achieve appropriate metabolic control and prevent the onset of future CVD in patients with T2DM.

## Abbreviations

ALT: alanine aminotransferase
AST: aspartate aminotransferase
BaPWV: brachial-ankle pulse wave velocity
BDI: beck depression inventory
BDHQ: brief, self-administered diet history questionnaire
BMI: body mass index
BP: blood pressure
CVD: cardiovascular disease
eGFR: estimated glomerular filtration rate
γ-GTP: γ-glutamyl transpeptidase
HDL: high-density lipoprotein-cholesterol
IPAQ: International physical activity questionnaire
MEQ: morningness-eveningness questionnaire
PSQI: Pittsburg sleep quality index
T2DM: type 2 diabetes mellitus
UAE: urinary albumin excretion
